# Dietary Fructose and Palmitic Acid Induce Shared and Divergent Transcriptional Responses in the Larval Midgut of *Drosophila melanogaster*

**DOI:** 10.3390/cimb48030313

**Published:** 2026-03-14

**Authors:** Laura Castañeda-Partida, Myriam Campos-Aguilar, Luis Felipe Santos-Cruz, Lizbeth Abigail Piña-Soto, Santiago Cristobal Sigrist Flores, María Eugenia Heres-Pulido, Irma Elena Dueñas-García, Elías Piedra-Ibarra, Rafael Jiménez-Flores, Alberto Ponciano-Gómez

**Affiliations:** 1 Toxicología Genética, Biología, Facultad de Estudios Superiores Iztacala, Universidad Nacional Autónoma de México, Los Barrios N° 1, Los Reyes Iztacala, Tlalnepantla 54090, Mexico; 2 Laboratorio de Inmunología (UMF), Facultad de Estudios Superiores Iztacala, Universidad Nacional Autónoma de México, Los Barrios N° 1, Los Reyes Iztacala, Tlalnepantla 54090, Mexico; 3 Escuela Nacional de Ciencias Biológicas, Instituto Politécnico Nacional, Av. Wilfrido Massieu 399, Nueva Industrial Vallejo, Gustavo A. Madero, Mexico City 07700, Mexico; 4 Fisiología Vegetal (Unidad de Biología Tecnología y Prototipos), Facultad de Estudios Superiores Iztacala, Universidad Nacional Autónoma de México, Los Barrios N° 1, Los Reyes Iztacala, Tlalnepantla 54090, Mexico

**Keywords:** *Drosophila melanogaster*, midgut, transcriptomics, fructose, palmitic acid, dietary stress, nutrient integration

## Abstract

Background: High-energy diets enriched in simple sugars and saturated fatty acids alter metabolic homeostasis, yet how distinct nutrients are integrated at the transcriptional level remains incompletely understood. Methods: Here, we profiled the larval midgut transcriptome of *Drosophila melanogaster* following 24 h exposure to diets enriched with 5% fructose (FD), 1% palmitic acid (PD), or their combination (MD). RNA sequencing (Illumina NovaSeq) was performed on pooled third-instar larval midguts, and differential expression analyses were conducted to identify diet-associated transcriptional changes. Results: The results revealed extensive transcriptional remodeling, with most responses being diet-specific, alongside a conserved core of genes regulated across all treatments. Shared transcriptional signatures were associated with proteostasis and amino acid transport pathways. Comparative and pattern-based analyses further uncovered discordant gene sets and pathway enrichments that were unique to individual diets or to the combined exposure. Notably, the mixed diet induced distinct expression patterns with specific functional signatures that were not predictable from either nutrient alone. Conclusions: Together, these findings indicate that the larval midgut integrates carbohydrate and lipid inputs through coordinated and context-dependent transcriptional responses, highlighting the importance of nutrient combinations in shaping epithelial metabolic programs.

## 1. Introduction

Excess consumption of sugar and saturated fat in modern Western diets has become a major driver of metabolic diseases worldwide [1,2]. Ultra-processed foods, often enriched with fructose (e.g., high-fructose syrups) and palmitic acid (the predominant saturated fatty acid in palm oil), are inexpensive and highly palatable, leading to overconsumption [2]. Epidemiological studies link these dietary components to obesity, type 2 diabetes, cardiovascular disease, and other chronic non-communicable diseases [1,2]. For instance, high-sugar diets can induce metabolic dysregulation—causing insulin resistance and adiposity—which predispose to diabetes [1]. Likewise, high intake of saturated fats correlates with dyslipidemia and systemic inflammation, compounding metabolic risk [3]. Notably, emerging evidence also implicates Western diets in neurological dysfunction: diets rich in fructose and palmitate have been associated with elevated risk of depression and even accelerated onset of neurodegenerative conditions [2]. These findings underscore that diet-induced disturbances are multi-systemic, affecting metabolic and functional homeostasis across tissues.

To unravel how nutrient-rich diets perturb organismal biology, researchers have turned to model organisms and omics approaches. *Drosophila melanogaster* has emerged as a powerful system to study nutritional genomics, thanks to its conserved metabolic pathways and ~75% gene homology to humans [4]. Flies offer practical advantages (short lifecycle, low cost) and have been extensively used to dissect gene–diet interactions at the whole-organism level [5]. Crucially, *Drosophila* exhibits mammalian-like responses to dietary excess including weight gain, hyperglycemia, and insulin resistance making it a relevant model for diet-induced gene regulation [1]. Numerous transcriptomic studies in flies demonstrate that altering macronutrient content triggers broad changes in gene expression [2]. For example, Stobdan et al. (2019) showed that a high-fat diet in *Drosophila* upregulated genes involved in lipid metabolism and storage homeostasis, relative to a normal diet [6]. Intriguingly, that study also noted increased expression of circadian-related genes linked to excessive feeding behavior on the high-fat regimen [2]. Similarly, Rivera et al. (2019) reported that adding saturated fat to a fly diet altered expression of genes controlling memory, olfaction, and motor functions in the head [7]. Many of these fly genes map to pathways for neuronal signaling and metabolism, suggesting that dietary lipids can influence brain gene networks relevant to cognitive health [2]. Together, fly models highlight that dietary fructose and fat provoke genome-wide transcriptional reprogramming, affecting pathways from energy metabolism to neural function.

Concordant insights come from mammalian models. In rodents, high-fructose diets reliably induce features of metabolic syndrome insulin resistance, visceral adiposity, hypertension, and fatty liver even in the absence of excess caloric intake [1,8]. Mechanistic studies show that fructose can directly alter hepatic gene expression, tipping the balance toward lipogenesis over fat oxidation [9]. For instance, excessive fructose intake drives up regulation of lipogenic enzymes in the liver and promotes triglyceride accumulation, fostering non-alcoholic fatty liver disease (NAFLD) [9]. High-fructose feeding also increases expression of intestinal fructolytic genes (e.g., ketohexokinase) as an adaptive response to load [10]. In parallel, diets enriched in palmitic acid (a common saturated fatty acid) elicit pro-inflammatory and metabolic gene expression changes. Mice on a palmitate-rich high-fat diet exhibit elevated inflammatory cytokines and macrophage activation in metabolic tissues, partly via toll-like receptor signaling and epigenetic reprogramming of immune cells [3]. Notably, a recent study showed that chronic palmitate intake can induce “innate immune memory” a long-lasting hyper-inflammatory state by altering bone marrow stem cell chromatin landscapes [3]. Such saturated fat-driven transcriptional reprogramming may underlie the low-grade inflammation and insulin resistance observed in obesity. Beyond peripheral metabolism, saturated fats can also affect gene expression in the brain. Schommer et al. (2018) demonstrated that a palmitic acid-enriched diet increased mRNA and protein levels of tyrosine hydroxylase (the dopamine synthesis enzyme) and α-synuclein in mouse brain [11]. This suggests that high-palmitate diets could modulate neurotransmitter pathways, potentially linking dietary fat to neurodegenerative disease risk [11]. Thus, evidence from mammals aligns with *Drosophila* findings: diets high in fructose or palmitate systematically rewire metabolic and signaling pathways at the transcriptomic level.

Multi-tissue transcriptomic profiling and functional analyses reinforce several recurring themes in diet-induced gene regulation. One common signature is disruption of nutrient metabolism pathways. For example, a recent fly RNA-sequencing study found that a combined fructose/palmitate diet significantly downregulated genes for essential and non-essential amino acid biosynthesis in both midgut and brain tissues [2]. The reduced expression of enzymes in branched-chain amino acid and glutamate synthesis pathways may reflect a feedback response to excess dietary sugar and fat, potentially affecting neurotransmitter availability and gut nutrient sensing [2]. In the same study, genes involved in mitochondrial oxidative phosphorylation were upregulated under the fructose–palmitate diet, suggesting a compensatory increase in energy expenditure pathways [2]. Other groups have observed that high-sugar diets activate stress and detoxification genes (e.g., purine catabolism enzymes) in flies, paralleling changes in human metabolism [1]. Indeed, metabolomic data from human cohorts reveal that dietary sugar intake strongly correlates with circulating purine levels [1], mirroring the uric acid accumulation seen in sugar-fed *Drosophila* [1]. Collectively, these cross-species findings illustrate how nutrient-excess diets can reshape gene expression networks that govern metabolism, immunity, and even neural processes.

In summary, contemporary research from *Drosophila* to mammals indicates that fructose- and palmitate-rich diets provoke widespread transcriptomic shifts with tangible functional consequences for organismal physiology. High sugar and saturated fat intake not only disrupt metabolic homeostasis promoting fat storage, insulin resistance, and inflammation but also perturb signaling pathways and gene networks critical for maintaining health. Transcriptomic and functional studies are therefore crucial to map the molecular underpinnings of diet-induced phenotypes beyond overt metabolic endpoints. By integrating evidence from multiple model systems, the present study seeks to advance our understanding of how fructose- and palmitic acid-enriched diets reprogram biological pathways.

## 2. Materials and Methods

### 2.1. Drosophila Rearing and Experimental Design

Wild-type *D. melanogaster* Canton-S larvae were reared under standard conditions and transferred to their respective experimental diets at 72 ± 4 h post-hatching. Third-instar larvae (96 ± 4 h) were collected after 24 h of dietary exposure. To minimize parental epigenetic carryover, the breeding stock was maintained for three generations on the standard diet before initiating experimental treatments.

### 2.2. Diet Preparation

The standard diet formulation contained 2.6% yellow cornmeal, 4% inactive dry yeast, 2% agar, 3% sucrose, 1.5% methylparaben, and 0.3% propionic acid per 100 mL mixture. Experimental diets were prepared by adding 1% palmitic acid, 5% fructose, or both to the same base formulation. The selection of these concentrations was guided by previous nutritional studies in *Drosophila*. Hemphill et al. investigated diets enriched in coconut oil and sucrose, whereas Wang et al. and Shi et al. evaluated palmitic acid at concentrations of 2% and 3%, respectively [12,13,14]. Based on these reports, preliminary experiments were conducted from eclosion to pupation using ND, palmitic acid (3.2%), and fructose (5%) diets, with five replicate tubes per treatment and one pair of flies per replicate. These tests indicated that reducing palmitic acid to 1% avoided alterations in emergence relative to the control diet while preserving the physicochemical consistency of the culture medium [12,13,14]. All diets were freshly prepared, mixed at 60 °C until homogeneous, and cooled before use.

### 2.3. Experimental Diets

Four dietary conditions were used to examine the impact of nutritional composition on midgut gene expression in *Drosophila melanogaster* larvae: a standard control diet, a 1% palmitic acid-enriched diet (PD), a 5% fructose-enriched diet (FD), and a mixed diet (MD) containing both 1% palmitic acid and 5% fructose. For each condition, midgut tissues were collected from pooled samples of 100 third-instar larvae per replicate and processed following a comparative transcriptomic design.

### 2.4. Midgut Dissection and RNA Extraction

Midguts were dissected from third-instar larvae (96 ± 4 h) using cold 1× PBS (4 °C). A total of 100 midguts biological replicate were collected within 30 min and immediately stored in TRIzol reagent (Invitrogen, Waltham, MA, USA) at −70 °C. Total RNA was extracted using the RNeasy Kit (QIAGEN, Germantown, MD, USA) according to the manufacturer’s instructions, followed by mRNA purification with the RNeasy Pure mRNA Bead Kit (QIAGEN). RNA quality was assessed using a NanoDrop 200 spectrophotometer (Thermo Scientific, Waltham, MA, USA), ensuring OD_260_/_280_ ≈ 2.0 and concentrations ≥ 5 ng/µL, and RNA integrity was evaluated using an Agilent Fragment Analyzer (Agilent technology, Santa Clara, CA, USA), with samples meeting the required RNA quality criteria (RIN/RQN ≥ 6–7) for library preparation and sequencing. For each dietary condition, two independent biological replicates were analyzed. Each replicate consisted of an independent pool of 100 larval midguts collected separately from which one RNA-seq library was generated. In total, two independent RNA-seq libraries per condition were sequenced. No technical replicates were included.

### 2.5. RNA Sequencing and Data Processing

RNA sequencing was performed by Novogene (Sacramento, CA, USA) on the Illumina NovaSeq 6000 platform, generating paired-end reads in FASTQ format. Quality filtering and adapter trimming were carried out using fastp (v0.23.2), and read quality metrics (Q20, Q30, GC content) were computed for all samples. Clean reads were aligned to the *Drosophila melanogaster* reference genome (assembly id = 204923) using HISAT2 (v2.2.1). Transcript assembly was conducted with StringTie v3.0.3, and comparison against reference annotation files was performed using GffCompare v0.12.10. Gene-level read counts were obtained with featureCounts v2.0.2, and expression levels were normalized as RPKM values, accounting for sequencing depth and transcript length.

### 2.6. Differential Expression Analysis

Differentially expressed genes (DEGs) were identified using the DESeq2 package (Bioconductor, v1.40.2). Comparisons were made between each treatment (FD, PD, MD) and the control. Genes with adjusted *p* < 0.05 (Benjamini–Hochberg FDR) and |log_2_FC| ≥ 1 were classified as significantly differentially expressed.

Intersection analyses were then performed to identify genes shared among the three treatments (FD, PD and MD). Shared up- or downregulated sets were used for subsequent visualization and enrichment analyses.

### 2.7. Functional Enrichment and Network Analyses

Functional enrichment was conducted for each DEG set using Gene Ontology (GO) Biological Process (BP) terms. Analyses were performed in R (v4.3.1) using the packages clusterProfiler (v4.6.2), org.Dm.eg.db, enrichplot, and ggplot2. Significance was assessed using Fisher’s exact test with FDR correction (q < 0.05).

For visualization, the top 15 most significant GO terms per group (up and down) were plotted as bipartite networks: left nodes represented GO processes, right nodes represented associated genes, and edges indicated functional connections. Node size was proportional to the number of mapped genes, and color intensity reflected −log_10_ FDR values. Terms lacking edges were excluded to emphasize predominant interactions. All bipartite visualizations were generated in Python (v3.11) using pandas, matplotlib, networkx, and adjustText.

### 2.8. Concordant and Discordant Expression Patterns

Shared genes across all diets were classified according to the direction of their log_2_FC values, resulting in four discordant patterns: ↑↑↓, ↑↓↓, ↓↑↑, and ↓↓↑. Pattern-based subsets were used for comparative and topological analyses.

A horizontal heatmap was generated to depict fold-change intensity across the three treatments, with columns representing genes and rows corresponding to fructose, palmitic acid, and mixed diets. Color gradients ranged from blue (downregulation) to red (upregulation).

Functional enrichment of each discordant subset was performed as described above, restricted to GO Biological Process terms. Networks were visualized separately for each pattern to reveal the predominant biological themes. All analyses and plots were executed in Python (v3.11) using pandas, numpy, matplotlib, seaborn, and network.

To evaluate whether the magnitude of regulation among genes shared across all treatments differed between diets, we performed an exploratory paired non-parametric Friedman test on DESeq2 log_2_FC estimates for the shared upregulated and shared downregulated DEG sets (each gene treated as a matched block across FD, PD, and MD). This analysis was used to describe overall trends in fold-change magnitude and does not replace model-based inference; differential expression calls were based exclusively on DESeq2 applied to raw counts with its internal normalization and variance estimation.

### 2.9. Validation of Gene Expression by RT-qPCR

Expression levels of Hsp70 were assessed by quantitative reverse transcription PCR (RT-qPCR) as an independent transcriptional readout. cDNA was synthesized from total RNA using the High-Capacity cDNA Reverse Transcription Kit (Applied Biosystems, Foster City, CA, USA). Amplification was performed with SYBR Green Master Mix (Thermo Fisher Scientific, Waltham, MA, USA) under standard cycling conditions. Primer efficiency was verified by standard curves, and relative expression was calculated by the 2^−ΔΔCt^ method using Actin as the reference gene. Results are expressed as mean ± SD from three independent biological replicates, each analyzed in technical triplicate, and statistical significance was assessed by one-way ANOVA followed by Tukey’s post hoc test (* *p* < 0.05, ** *p* < 0.01, *** *p* < 0.001).

## 3. Results

### 3.1. Comparative Transcriptomic Profiling of the Larval Midgut Across Dietary Conditions

Transcriptomic profiling of the larval midgut revealed extensive gene expression remodeling induced by the three experimental diets: fructose, palmitic acid, and the combined (mixed) diet. Relative to the standard condition, fructose supplementation upregulated 738 genes and downregulated 728, while the palmitic acid diet altered 2174 genes with a balanced direction of change. The mixed diet produced an intermediate response, with 1973 differentially expressed genes, including 980 upregulated and 993 downregulated (Supplementary Figure S1), including distinct yet overlapping transcriptional adaptations to each dietary component.

**Figure 1:**
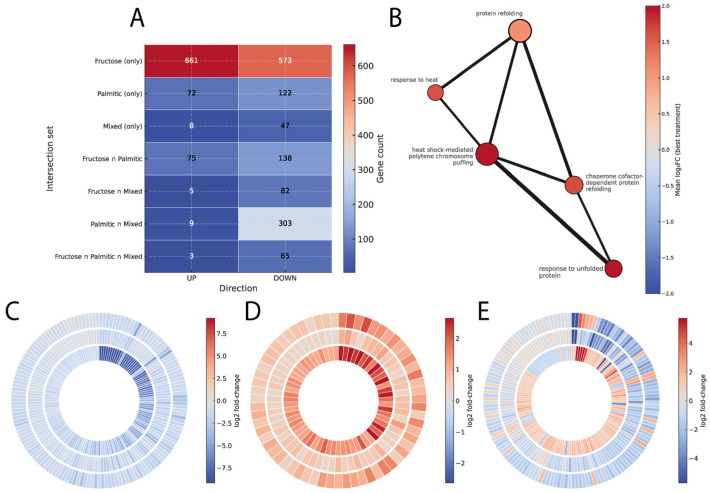
Comparative integration of differentially expressed genes across treatments. Panel (**A**) shows a heatmap depicting the number of exclusive and shared genes among the datasets derived from each treatment and their direction of change. Panel (**B**) displays the network of enriched biological terms associated with the intersection of shared genes, where the thickness of the edges reflects functional overlap. Panels (**C**–**E**) present radial diagrams summarizing the significant genes shared across the three comparisons: each ring corresponds to a treatment (inner = Fructose, middle = Palmitic acid, outer = Mixed), and the color scale represents log_2_ fold change, with red tones indicating upregulation and blue tones downregulation. Panel (**C**) groups genes that decrease consistently across all three treatments, panel (**D**) shows those that increase concordantly, and panel (**E**) includes genes with discordant patterns, where one or more treatments display an opposite direction of change. The symbol ∩ indicates the intersection of gene sets shared between the indicated treatments.

Comparison of differential expression sets revealed that most genes were uniquely responsive to a single diet, whereas smaller subsets were shared between fructose and palmitate or across all three treatments (Figure 1A). The latter group indicates the presence of conserved nutrient-sensitive pathways engaged by different metabolic contexts.

Functional enrichment of the shared genes showed a coherent network of biological processes related to protein folding and stress response (Figure 1B). Prominent nodes included protein refolding, response to heat, and chaperone cofactor-dependent protein folding, consistent with activation of proteostasis and cytoprotective programs. The full enrichment map, encompassing both up- and downregulated terms, is provided in Supplementary Figure S2.

To further explore expression directionality, a radial analysis of mean fold-change values was applied to the genes common to all diets (Figure 1C–E). Three patterns emerged: 147 genes exhibited consistent downregulation, 63 genes showed uniform upregulation, and 144 displayed discordant regulation increasing in one or two diets while decreasing in the others. This pattern illustrates shared regulatory pathways but varying response magnitudes, suggesting that the combined exposure to fructose and palmitic acid fine-tunes the transcriptional response rather than simply amplifying it.

### 3.2. Common Downregulated Genes and Associated Metabolic Networks

**Figure 2:**
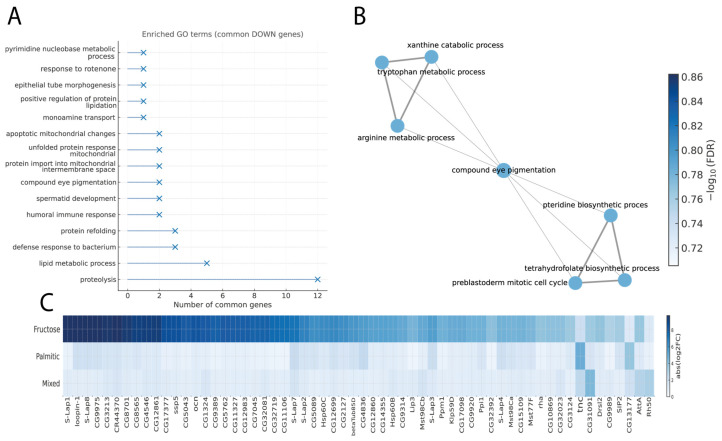
Biological processes and expression patterns of genes commonly downregulated across the three experimental diets. Panel (**A**) presents enriched GO Biological Process terms for commonly downregulated genes under fructose, palmitic acid, and mixed diets. The *x*-axis indicates the number of genes per term, and marker size reflects the number of genes associated with each GO term. Panel (**B**) depicts the GO subnetwork, with nodes representing biological processes and edges indicating shared genes; node size corresponds to gene count and color intensity (Blues scale) to adjusted significance (−log_10_ FDR). Panel (**C**) displays a horizontal heatmap of 60 shared downregulated genes, showing log_2_ fold change (log_2_FC) values across treatments. Rows correspond to diets and columns to genes, with darker blues indicating greater decreases in expression.

The comparative gene expression analysis identified 354 significantly downregulated genes shared among the three experimental treatments: fructose, mixed, and palmitic acid. Functional enrichment of these genes revealed that the most representative biological processes were mainly related to metabolic and degradative pathways (Figure 2A). The five processes with the highest number of genes were proteolysis (12 genes), lipid metabolic process (5 genes), defense response to bacterium (3 genes), protein refolding (3 genes), and humoral immune response (2 genes), followed by spermatid development (2 genes). These data show a shared decrease in genes associated with catabolic and cellular defense mechanisms across all diets.

In the biological network (Figure 2B), xanthine catabolic, arginine metabolic, and pteridine biosynthetic processes formed an interconnected functional core connections involving pathways related to compound eye pigmentation.

The comparison of expression magnitude among treatments (Figure 2C) showed that the strongest downregulation occurred under fructose supplementation, with genes such as S-Lap1 (−10.84, −1.20, −0.68), loopin-1 (−10.30, −1.10, −2.01), and S-Lap8 (−10.08, −1.08, −2.00) exhibiting the largest negative log_2_ fold-change values. Under palmitic acid exposure, genes including tnc (−2.10, −2.66, −4.93) and CG13177 (−1.31, −2.00, −3.84) showed the strongest repression, while the mixed diet preferentially affected genes such as CG31091 (−3.62, −4.43, −1.63) and Rh50 (−1.44, −3.20, −0.73). Altogether, these data describe a consistent downregulation pattern across the three dietary conditions, with the magnitude of repression being strongest under fructose and intermediate under palmitic acid and mixed treatments.

### 3.3. Common Upregulated Genes and Associated Transport Networks

**Figure 3:**
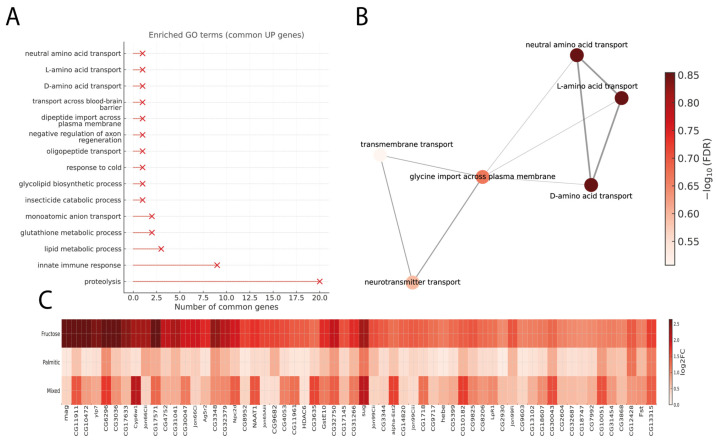
Biological processes and expression patterns of genes commonly upregulated across the three experimental diets. Panel (**A**) presents enriched GO Biological Process terms for genes commonly upregulated genes under the three treatments. The *x*-axis denotes the number of genes per term, and marker size reflects the number of genes associated with each GO term. Panel (**B**) shows the GO subnetwork, where nodes correspond to biological processes and edges to shared genes; node size represents mapped gene count and color intensity (Reds scale) adjusted significance (−log_10_ FDR). Panel (**C**) shows a horizontal heatmap of 60 shared upregulated genes, displaying log_2_FC values across fructose, palmitic, and mixed diets. Rows indicate treatments and columns genes, with darker reds representing greater expression increases.

The analysis of enriched GO terms among the commonly upregulated genes revealed 13 functional categories, the most representative being proteolysis (20 genes), innate immune response (9 genes), and lipid metabolic process (3 genes), followed by glutathione metabolic process and monoatomic anion transport (2 genes each). Additional enriched processes included insecticide catabolic process, glycolipid biosynthetic process, response to cold, oligopeptide transport, negative regulation of axon regeneration, dipeptide import across plasma membrane, and transport across blood–brain barrier (1 gene each) (Figure 3A).

The network representation (Figure 3B) showed a strong connection among transport-related processes, with neutral amino acid transport, L-amino acid transport, and D-amino acid transport forming a tightly associated cluster. These terms were also functionally linked with glycine import across plasma membrane and neurotransmitter transport, forming a clustered representation of transport-related GO terms.

In terms of expression magnitude (Figure 3C), genes such as CG6296 (3.09, 1.68, 1.00), CG11911 (2.94, 1.46, 0.58), and mag (2.85, 0.45, 0.24) exhibited the largest differences among treatments, with transcriptional activity consistently higher under the fructose condition, followed by the mixed and palmitic acid diets, respectively. These profiles show a graded increase in fold change across treatments, with fructose consistently showing the strongest transcriptional upregulation among the shared genes.

### 3.4. Differential Magnitude of Common Gene Regulation Among Treatments

To explore whether the three dietary conditions (FD, PD and MD) elicited quantitative differences in the magnitude of shared gene expression changes, we applied an exploratory paired Friedman test to DESeq2 log_2_FC estimates (treatment vs. control) for genes shared across all diets, treating each gene as a matched block across FD, PD, and MD. In the set of commonly repressed genes, the Friedman test revealed significant overall differences among treatments (χ^2^ = 7.8, *p* = 0.019). Pairwise comparisons showed that the fructose diet was associated with more pronounced repression than the palmitic acid or mixed diets, with mean Δlog_2_FC differences of –0.37 (95% CI [−0.58, −0.14]; *p* = 0.018) versus the mixed diet and −0.29 (95% CI [−0.52, −0.07]; *p* = 0.032) versus the palmitic acid diet. In contrast, the difference between palmitic acid and mixed diets was smaller and not significant (Δ = −0.11; 95% CI [−0.33, +0.08]; *p* = 0.226). These results are presented as a summary of fold-change trends among the shared gene set; gene-wise differential expression inference was based on DESeq2.

In the set of commonly induced genes, neither the Friedman test (χ^2^ = 1.92, *p* = 0.38) nor the pairwise comparisons showed significant differences among treatments. Mean expression differences were small and their confidence intervals crossed zero: +0.19 (95% CI [−0.04, +0.38]; *p* = 0.134) for fructose versus mixed, +0.06 (95% CI [−0.16, +0.27]; *p* = 0.438) for palmitic acid versus mixed, and +0.09 (95% CI [−0.11, +0.28]; *p* = 0.362) for fructose versus palmitic acid. Altogether, these findings indicate that the magnitude of induction among shared genes was comparable across the three dietary conditions.

### 3.5. Discordant Expression Among Diets and Associated Functional Analysis

**Figure 4:**
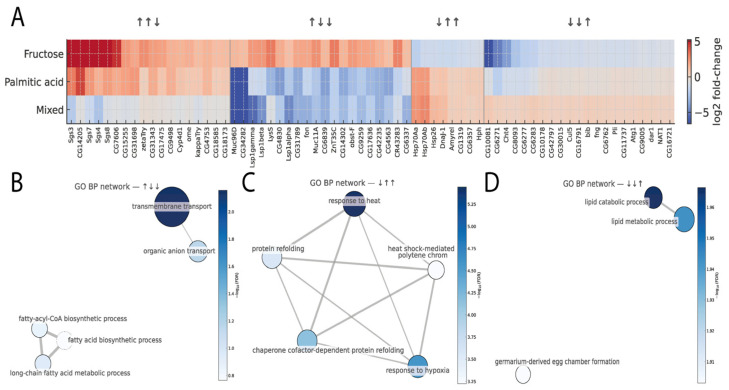
Discordant expression patterns and associated functional networks across dietary treatments. Panel (**A**) shows a horizontal heatmap of genes exhibiting discordant expression profiles across fructose, palmitic acid, and mixed diets (patterns ↑↑↓, ↑↓↓, ↓↑↑, and ↓↓↑). Color intensity represents log_2_ fold-change relative to the control, with blue and red tones indicating down- and upregulation, respectively. Panels (**B**–**D**) display GO Biological Process (BP) subnetworks derived from genes in each discordant category. Nodes correspond to enriched biological processes and edges to shared genes; node size indicates the number of mapped genes, and color intensity (Blues scale) reflects adjusted significance (−log_10_ FDR).

The analysis of genes with discordant expression among the three experimental diets (fructose, palmitic acid, and mixed) revealed four main patterns of change (↑↑↓, ↑↓↓, ↓↑↑, and ↓↓↑), which represent specific directions of relative regulation among treatments (Figure 4A). The ↑↑↓ pattern corresponds to genes increased under fructose and palmitic acid but reduced in the mixed diet; the ↑↓↓ pattern to genes upregulated only in fructose and downregulated in the other two; the ↓↑↑ pattern to genes repressed under fructose but increased in palmitic acid and mixed diets; and the ↓↓↑ pattern to genes repressed under fructose and palmitic acid but overexpressed exclusively in the mixed diet.

In total, 249 genes met the criteria of |log_2_FC| > 1 and FDR < 0.05, unevenly distributed among the four patterns, with 86 genes in ↑↑↓, 63 in ↑↓↓, 54 in ↓↑↑, and 46 in ↓↓↑. In the ↑↑↓ pattern, genes such as Sgs3, CG14205, Sgs7, Sgs4, and Sgs8 showed strong upregulation under fructose and palmitic acid with a marked decrease in the mixed diet. The ↑↓↓ pattern included genes such as Muc96D, Lsp1gamma, Lsp1beta, LysS, and CG4830, which are annotated with functions related to transport and metabolic processes. The ↓↑↑ pattern comprised genes related to heat response and proteostasis maintenance, including Hsp70Aa, Hsp70Ab, Hsp26, and DnaJ-1, which were activated under palmitic acid and mixed diets but repressed under fructose. Finally, the ↓↓↑ pattern contained fewer genes with an opposite trend, including CG11737, Atg1, CG9005, and dar1, which were preferentially induced under the mixed diet.

Functional enrichment analysis based on Gene Ontology Biological Process (GO BP) terms revealed that each pattern involved distinct biological routes (Figure 4B–D). In the ↑↓↓ pattern, significantly enriched terms clustered into a network centered on transmembrane transport, including organic anion transport and long-chain fatty acid biosynthetic and metabolic processes, with enrichment of terms related to transport and lipid metabolism. The ↓↑↑ pattern showed a coherent network associated with protein refolding, heat response, hypoxia response, chaperone cofactor-dependent protein folding, and heat shock mediated polytene chromosome puffing, comprising stress- and proteostasis-related GO terms. In contrast, the ↓↓↑ pattern displayed a smaller network dominated by lipid catabolism and lipid metabolic processes, along with germline-derived egg chamber formation, reflecting a link between metabolic remodeling and tissue differentiation mechanisms. The ↑↑↓ pattern did not yield significantly connected terms after FDR correction.

Among the genes identified in these sets, Hsp70 was assessed by RT-qPCR, showing differential expression across dietary conditions (Supplementary Figure S3).

## 4. Discussion

In this study, we used comparative transcriptomics to characterize how dietary fructose (FD), palmitic acid (PD) and their combination (MD) reshape gene expression in the *Drosophila melanogaster* midgut. Our results support three main observations. First, the midgut mounts a conserved core response to nutrient stress that is enriched for proteostasis and amino acid transport functions, consistent with common cellular coping strategies across nutrient classes. Second, palmitate elicits the most extensive transcriptional remodeling in terms of the number of differentially expressed genes and engages canonical lipid-associated responses (ER stress/unfolded protein response, mitochondrial and lipid metabolism, inflammatory signaling), whereas fructose produces a more targeted carbohydrate-centric program (sugar transport, carbohydrate metabolism, nutrient-sensing pathways). Third, combined exposure produces a mixed profile with both additive and distinct features: MD is associated with pathway activations (oxidative stress, autophagy/lysosomal function, reorganization of cellular transport) not observed from either single nutrient alone. Together, these observations indicate that intestinal epithelial responses to diet are both shared and nutrient-specific and that nutrient combinations can produce qualitatively distinct molecular states.

A cross-comparison of the transcriptional responses induced by the fructose-enriched, palmitic acid-enriched, and combined diets revealed extensive gene expression remodeling, with distinct gene sets associated with each condition and a smaller subset shared among treatments. This pattern indicates that, although each nutrient imposes particular metabolic challenges, there is a conserved core response that is activated independently of nutritional context. Within this shared subset, processes related to protein refolding and heat stress response were consistently observed, highlighting the recurrent enrichment of proteostasis-related pathways in the midgut transcriptome.

Previous studies have shown that diets rich in sugars or lipids can induce cellular stress and disrupt metabolic homeostasis, promoting compensatory mechanisms that include the activation of chaperones and protein maintenance systems [15]. Consistently, dietary exposure to high-energy nutrients has been reported to modulate intestinal epithelial integrity and the activity of proteins involved in cell turnover, particularly under metabolic overload conditions [16]. In addition, energy metabolism and the response to proteotoxic stress have been described as part of a functional axis in which the handling of misfolded proteins acts as a sensor of nutritional status [2]. Sustained disruption of epithelial homeostasis has also been linked to tissue vulnerability and reduced adaptive capacity to dietary fluctuations [17]. Finally, studies under chronic high-fat diet conditions have shown that progressive loss of proteostasis contributes to physiological decline and reduced lifespan, providing a broader context for the proteostasis-related transcriptional signatures identified in this study [18].

Taken together, these findings support the interpretation that the coordinated activation of chaperones and protein refolding pathways observed in our data is consistent with an adaptive transcriptional response associated with the maintenance of intestinal epithelial functionality under the metabolic load imposed by the diets. The presence of genes regulated in discordant directions among treatments suggests that the combination of fructose and palmitic acid does not necessarily produce an simple combined response, but rather a more refined modulation of the proteostatic balance. This supports the view that the integration of nutritional signals in the epithelium is complex, and that the resulting metabolic state depends on the interaction between stress pathways, protein handling, and energy availability.

The comparative analysis of genes consistently downregulated across the fructose, palmitic, and mixed diets revealed a shared repression of metabolic and degradative pathways, consistent with coordinated transcriptional adjustments in pathways related to cellular resource handling under nutritional excess. The enrichment of processes related to proteolysis, lipid metabolism, humoral immunity, and bacterial defense is compatible with a transcriptional downregulation of genes associated with catabolic functions and basal immune processes when challenged by high-energy diets. The metabolic network structure showed a convergence of xanthine, arginine, pteridine, and flavin biosynthetic processes, forming a functional core linked through shared cofactor dependencies. This organization supports the interpretation that the shared transcriptional response involves coordinated regulation of pathways related to nitrogen-containing compounds and redox-active metabolic intermediates.

Previous work has shown that diets enriched in sugars can induce systemic metabolic stress and reprogram lipid handling pathways, including shifts between lipid storage and β-oxidation [19]. Additionally, high-carbohydrate diets have been associated with alterations in purine metabolism and uric acid accumulation, linking nutrient excess to systemic metabolic load and tissue vulnerability [1]. High-fat diets, in turn, have been reported to modulate gut microbiota composition and induce inflammatory signaling cascades, illustrating that nutrient composition directly influences epithelial immune tone [20]. Moreover, variation in the quality of dietary fats has been shown to differentially reshape gene expression programs related to lipid metabolism and cellular maintenance, reinforcing the concept that dietary components exert selective pressure on metabolic regulatory networks [21]. Together, these studies support the view that dietary overload is associated with reorganization of metabolic and proteostatic pathways, even though the specific molecular outcomes depend on the nutrient source.

Taken together, the shared downregulation signature identified in our dataset is consistent with a transcriptional shift associated with reduced metabolic resource expenditure under sustained nutritional stress, with the intestine adjusting its balance between catabolism, redox metabolism, and immune readiness. The observation that the fructose treatment consistently produced the strongest repression, with the mixed diet displaying intermediate effects, supports the interpretation that nutritional signal integration is distinct, with carbohydrate-rich conditions associated with a stronger transcriptional impact on shared metabolic pathways.

The shared transcriptional upregulation pattern observed across the three dietary conditions revealed a convergence toward processes associated with protein turnover, amino acid handling, and lipid-related pathways. Notably, the strong enrichment of transport-related categories, particularly those associated with neutral, L- and D-amino acid transport, is consistent with transcriptional modulation of pathways related to substrate transport and availability. The graded expression differences among treatments, with fructose consistently inducing the strongest transcriptional activation and the mixed diet yielding intermediate values, support the view that different nutrient sources are associated with distinct transcriptional impacts that are integrated at the level of shared regulatory programs.

Previous work has shown that amino acid transport systems act as central sensors linking nutrient availability to systemic metabolic state, regulating growth, energy expenditure, and the balance between storage and utilization [22]. In addition, dietary shifts have been reported to restructure amino acid handling and metabolic routing, particularly through the modulation of transporter families involved in maintaining nutrient signaling and tissue-specific energy demand [23]. Tissue-level analyses in *Drosophila* have further demonstrated that the distribution and activity of amino acid transporters varies according to metabolic context, supporting the idea that transporter upregulation reflects targeted physiological adaptation rather than generalized stress [24]. Moreover, the regulation of amino acid uptake has been described as a key component of metabolic coordination across organ systems, coupling nutrient-derived signals to growth and energy allocation pathways [25]. Finally, dietary exposure to sugars and fatty acids has been shown to trigger distinct signaling responses that adjust nutrient perception and metabolic prioritization in a distinct manner [26].

Taken together, these observations are consistent with coordinated transcriptional activation of amino acid transport and associated metabolic pathways in response to dietary load. The differential relationship between fructose and palmitic acid treatments suggests that nutrient-derived signals interact in a distinct manner, rather than producing simple additive effects. This supports the interpretation that the transcriptional signatures identified here reflect a regulated integration at the transcriptional level of metabolic state, substrate availability, and nutrient-specific signaling inputs.

The comparative analysis of genes displaying discordant expression patterns among the three dietary treatments revealed four distinct directional profiles that are consistent with differential nutritional modulation rather than uniform transcriptional responses. These patterns (↑↑↓, ↑↓↓, ↓↑↑, and ↓↓↑) represent systematic differences in gene expression in which transcriptional activation or repression varies relative to each diet. Notably, the ↑↑↓ pattern, characterized by increased expression under fructose and palmitic acid but reduced levels in the mixed diet, comprised genes associated with salivary secretion and cuticular formation, but did not converge into a coherent functional group under the enrichment criteria applied. In contrast, the remaining three patterns exhibited well-defined biological organization.

The ↑↓↓ pattern showed an increase in expression only under the fructose-enriched diet, accompanied by systematic repression in the palmitic acid diet and in the mixed diet. The representative genes of this set were mainly associated with transmembrane transport and peptide catabolism. The functional enrichment analysis revealed a network centered on organic anion transport, carbohydrate transport, and long-chain fatty acid biosynthesis, consistent with a transcriptional program related to substrate handling and mobilization that is preferentially associated with carbohydrate exposure.

Studies in *Drosophila* have shown that SLC22 family transporters regulate the movement of metabolites, organic anions, and signaling compounds, acting as nodes that adjust inter-tissue communication and metabolic equilibrium under variations in energy availability [27]. This principle fits within a broader systemic framework in which OAT/OATP-related transporters function as integrators of metabolic state, modulating the flow of solutes in response to nutritional composition [28]. Additionally, in insects, multidrug and OATP-like transporters have been implicated in physiological adaptation to the ingestion of chemical compounds, reinforcing the idea of a flexible and context-dependent role for these transport systems [29]. Finally, the general metabolic framework in *Drosophila* indicates that carbohydrate utilization and the coupling toward lipogenic pathways is highly dependent on the relative availability of nutrients, supporting the concept that sugar-derived transcriptional signals may be differentially interpreted in the presence of high lipid availability [22].

Taken together, these elements are consistent with the ↑↓↓ pattern reflecting a transcriptional program preferentially associated with high carbohydrate intake, involving genes related to substrate redistribution and processing. The repression observed under palmitic acid and mixed diets suggests that the presence of fatty acids is associated with attenuation of this transcriptional program, altering the relative expression balance of genes linked to absorption, processing, and energy utilization. Thus, the metabolic integration between carbohydrates and lipids appears distinct at the transcriptional level, but depends on the relative proportion of energy sources, meaning that the signal derived from fructose is modulated or counterbalanced when the lipid input is dominant or equivalent.

The ↓↑↑ pattern showed repression under the fructose-enriched diet and increased expression under the palmitic acid-enriched and mixed diets. The most representative genes of this set included Hsp70Aa, Hsp26, and DnaJ-1, all associated with heat response, proteostasis maintenance, and chaperone-assisted protein folding. Functional analysis revealed a coherent network related to protein refolding, heat shock response, hypoxia response, chaperone cofactor-dependent folding, and chromosomal puffing induced by thermal stress, consistent with transcriptional activation of protein-handling pathways associated with increased metabolic load.

Previous studies in *Drosophila* have shown that systemic lipid mobilization is associated with protective cellular responses and with the modulation of protein homeostasis under physiological stress conditions [30]. Likewise, it has been documented that high-fat diets can alter acute stress tolerance, involving mechanisms dependent on chaperones and heat shock proteins [31]. Complementarily, it has been demonstrated that the transcriptional activity regulating Hsp70/Hsp26/Hsp68 genes is dynamic and context-dependent, where repressor factors can modulate their activation in response to cellular perturbations [32]. Together, these works provide a framework in which proteostasis-related transcriptional programs are dynamically modulated according to metabolic state and nutritional context.

In this context, the activation of the ↓↑↑ pattern under palmitic acid and mixed diet is consistent with a compensatory transcriptional response associated with protein integrity when lipid metabolism predominates, while its repression under fructose suggests that this response axis is not transcriptionally engaged in the same way when carbohydrates dominate the energy input. This indicates that the integration between lipids and proteostasis is complex at the transcriptional level and depends on the relative proportion of energy sources. Thus, the observed pattern can be interpreted as a lipid-associated proteostasis-related transcriptional program, whose expression is attenuated in a context where fructose is the main dietary source.

The ↓↓↑ pattern comprised a subset of genes that were downregulated under fructose and palmitic acid diets but upregulated exclusively in the mixed condition, indicating a transcriptional response that was specific to the combination of nutrients. Among the genes showing this trend, CG11737, Atg1, CG9005, and dar1 displayed the most pronounced differential expression. The functional enrichment analysis of this set revealed a compact network dominated by lipid catabolic and lipid metabolic processes, accompanied by terms associated with germline chamber formation, suggesting the coordinated involvement of metabolic remodeling pathways together with transcriptional programs linked to tissue differentiation.

Previous studies have established that the regulation of lipid metabolism in *Drosophila* is not limited to energy balance but participates in the definition of cellular states and in the coordination among metabolically active tissues [21]. It has been described that lipid mobilization and catabolism respond differentially to the composition and quality of the nutritional environment, generating specific transcriptional profiles that are distinct from those induced by each nutrient individually [30]. In addition, lipid metabolism is closely linked to programs of tissue organization and renewal in the intestine, where the integration of dietary signals has been associated with transcriptional programs related to structural maintenance and cellular dynamics [31]. Consistently, it has been observed that systemic adjustments in lipid trafficking and utilization may accompany states of tissue reorganization, supporting the view that lipid remodeling is associated with regulatory processes in cells and tissues with high physiological plasticity [32].

In this context, the ↓↓↑ pattern observed in our analysis can be interpreted as an distinct response derived from the simultaneous integration of lipid and carbohydrate sources, rather than as the independent effect of each diet separately. The exclusive activation of routes associated with lipid catabolism together with terms linked to tissue programs suggests that the combination of fructose and palmitic acid generates a particular metabolic state in the intestinal epithelium, capable of modulating the balance between lipid processing and structural reorganization. This response could reflect an adaptive adjustment that favors the functional stability of the tissue under conditions of complex nutritional load, in contrast to the more restrictive transcriptional profiles observed when a single type of nutrient predominates in the diet.

In sum, our findings show that fructose- and palmitic acid-enriched diets elicit broad but structured transcriptional remodeling in the *Drosophila* midgut, revealing both conserved and nutrient-specific regulatory signatures. The shared activation of proteostasis and amino acid transport pathways is consistent with a conserved transcriptional response associated with sustained metabolic load, whereas the discordant expression patterns demonstrate that the transcriptional responses to different nutrient sources vary depending on nutritional context. In particular, the mixed diet was associated with transcriptional states that differed from those observed under the individual fructose or palmitic acid diets, consistent with integrated regulation of lipid- and carbohydrate-responsive gene expression programs. These results highlight the importance of considering nutrient combinations, rather than single components, when evaluating the molecular impact of modern high-energy diets, and position the *Drosophila* midgut as a tractable model for dissecting the transcriptional logic of dietary stress integration.

## 5. Conclusions

Our findings show that fructose- and palmitic acid-enriched diets elicit broad but structured transcriptional remodeling in the *Drosophila* midgut, revealing both conserved and nutrient-specific regulatory signatures. The shared activation of proteostasis and amino acid transport pathways highlights a conserved transcriptional response associated with sustained metabolic load, whereas the discordant expression patterns demonstrate that transcriptional responses to different nutrient sources vary depending on nutritional context. In particular, the mixed diet was associated with transcriptional states that differed from those observed under the individual fructose or palmitic acid diets, reflecting integrated regulation of lipid- and carbohydrate-responsive gene expression programs. These results highlight the importance of considering nutrient combinations, rather than single components, when evaluating the molecular impact of modern high-energy diets, and position the *Drosophila* midgut as a tractable model for dissecting the systemic logic of dietary stress integration.

## Data Availability

Data are available on Figshare platform, under the link https://figshare.com/s/d6eeedde6ebed47d5ae4 (accessed on 12 March 2026).

## Supplementary Materials

The following supporting information can be downloaded at: https://www.mdpi.com/article/10.3390/cimb48030313/s1.
