# Supplementary material for: Dietary Fructose and Palmitic Acid Induce Shared and Divergent Transcriptional Responses in the Larval Midgut of Drosophila melanogaster
Source: Curr Issues Mol Biol. 2026 Mar 14;48(3):313. doi: 10.3390/cimb48030313 (PMC13025958; doi:10.3390/cimb48030313)
Supplement: Supplementary file 1 [file cimb-48-00313-s001.zip › cimb-4167464-supplementary.pdf]

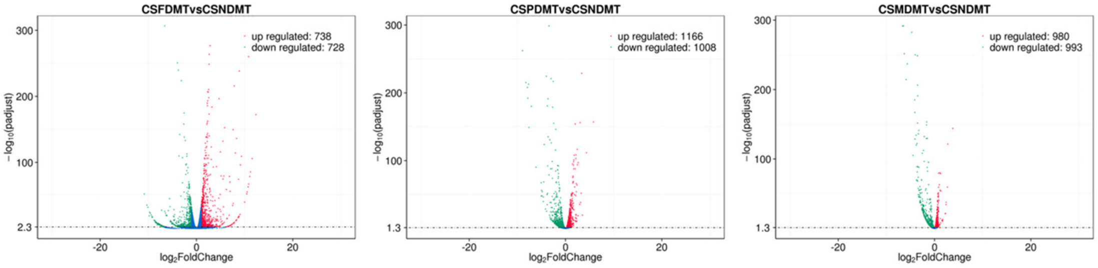

Supplementary Figure S1. Differential gene expression analysis in the larval midgut of *Drosophila melanogaster* under different diets. Volcano plots showing the distribution of differentially expressed genes in larvae fed diets enriched in fructose (left), palmitic acid (center), or a mixed diet (right), compared to the standard diet. The x-axis represents the $\log_2$ fold change in gene expression, while the y-axis indicates the adjusted significance value ($-\log_{10}$ p-adj). Red dots correspond to significantly upregulated genes, and blue dots represent significantly downregulated genes for each treatment.

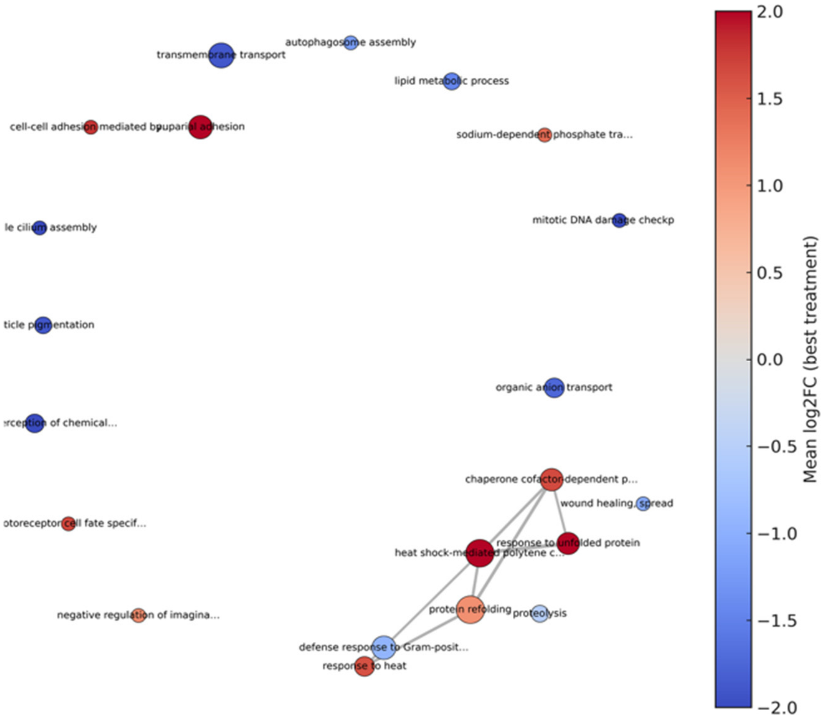

Supplementary Figure S2. Functional enrichment analysis of differentially expressed genes. Network map showing significantly enriched biological terms derived from differentially expressed genes in the larval midgut of *Drosophila melanogaster* under the three experimental diets. Each node represents a Gene Ontology (GO) term, and edges indicate semantic similarity between related terms. Node color reflects the average gene expression change (mean $\log_2$ fold change) within each term, with red tones indicating upregulation and blue tones indicating downregulation.

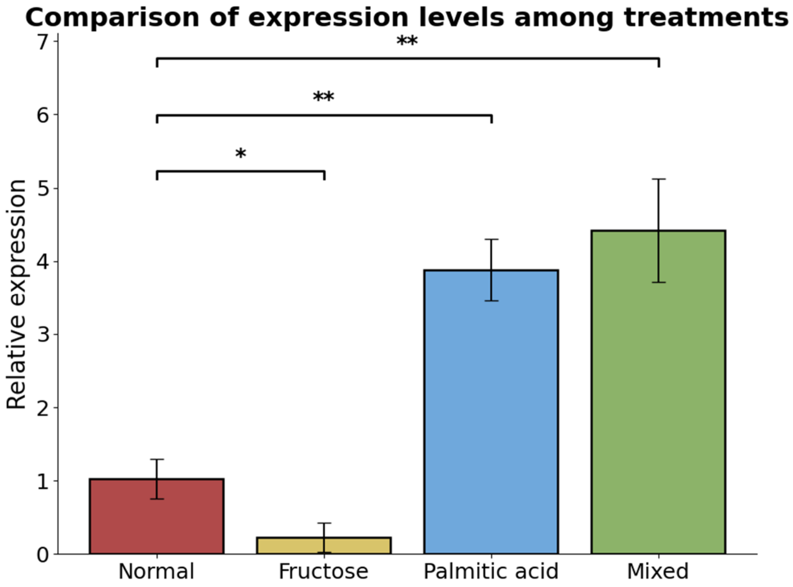

Supplementary Figure S3. Expression validation of Hsp70 under dietary treatments. Relative expression levels of *Hsp70* were quantified by RT-qPCR using RNA from larval midgut samples collected after exposure to fructose, palmitic acid, and mixed diets. *Actin* was used as the housekeeping gene for normalization. Bars represent mean ± SD from three independent biological replicates, and brackets with asterisks indicate significant differences compared with the control (* $p < 0.05$, ** $p < 0.01$, *** $p < 0.001$).
